# Continuous Wrist‐Derived Heart Rate Correlates With mYPAS Scores and Detects Between‐Assessment Heart‐Rate Elevations in Children: A Feasibility Study

**DOI:** 10.1002/pan.70309

**Published:** 2026-09-10

**Authors:** Shawn D. Safford, Sikiru Adewale, Denis Gracanin

**Affiliations:** ^1^ Division of Pediatric Surgery UPMC Children's Hospital Harrisburg Harrisburg Pennsylvania USA; ^2^ Department of Surgery University of Pittsburgh School of Medicine Pittsburgh Pennsylvania USA; ^3^ Department of Computer Science Virginia Tech Blacksburg Virginia USA

**Keywords:** continuous monitoring, digital health, heart rate, mYPAS, pediatric anesthesia, preoperative anxiety, wearable biosensors

## Abstract

**Background:**

Perioperative anxiety affects most children undergoing surgery and predicts emergence delirium, postoperative pain, and behavioral change. The validated modified Yale Preoperative Anxiety Scale (mYPAS) is scored at only a few discrete timepoints, leaving physiologic status between assessments unmeasured.

**Aims:**

We tested whether continuous wearable heart rate correlates with the mYPAS where scored, and whether heart‐rate elevations of undetermined cause occur during intervals without concurrent mYPAS observation.

**Methods:**

Children aged 4–17 undergoing elective surgery wore a wrist biosensor (Empatica EmbracePlus) from holding through induction; the mYPAS was scored at consent, parental separation, and induction. We assessed heart rate–mYPAS concordance and the proportion of heart‐rate elevations falling outside ±5 min of any mYPAS timepoint, stratified by premedication.

**Results:**

Of 32 consented children, 15 were analyzable (median age 10 years, nine male, eight premedicated). Induction‐window heart rate correlated with induction anxiety (Spearman *ρ* = 0.55, 95% CI 0.01–0.84, *p* = 0.034) and rose into induction in 13 of 15 children (median + 10 beats/min, *p* = 0.022). Of 90 heart‐rate elevations among 14 children with reliable timing, 71% fell outside the assessment windows (95% CI 58%–80%); 12 of 14 (86%) had ≥ 1 event outside all assessment windows. This pattern persisted regardless of premedication. Heart rate tracked children's state self‐report (*ρ* = 0.62, ages ≥ 9) but not trait anxiety; informants agreed modestly.

**Conclusions:**

In this small feasibility cohort, wrist‐derived induction‐window heart rate was associated with concurrent mYPAS scores. The majority of heart‐rate elevations of undetermined cause occurred outside scheduled mYPAS assessment windows. These exploratory findings require confirmation in a larger study incorporating raw physiologic signals, motion measurement, and continuous or event‐linked behavioral assessment, which would establish whether continuous physiologic monitoring can complement intermittent behavioral assessment by identifying physiologic events occurring between scheduled anxiety assessments.

## Clinical Implications

**What Is Already Known?:**

Pediatric perioperative anxiety is assessed at fixed timepoints with the mYPAS, which cannot capture stress occurring between those assessments.

**What New Information This Study Adds?:**

Continuous wrist heart rate agreed with the mYPAS at the moments it is scored, yet showed that most heart‐rate elevations occurred between those scheduled assessments, quantifying the proportion of elevations that occurred outside the scheduled assessment windows.

## 1 Introduction

Anxiety is the rule rather than the exception for children facing surgery, affecting more than 60% of pediatric surgical patients [1], and it is not benign: heightened preoperative distress predicts emergence delirium [2, 3], greater postoperative pain and analgesic use, prolonged recovery, and maladaptive behavioral changes that can persist for weeks [4, 5]. The clinical response—pharmacologic anxiolysis, child‐life support, parental presence—is effective when applied at the right moment. The difficulty is knowing when that moment is.

Current practice infers a child's anxiety from the modified Yale Preoperative Anxiety Scale (mYPAS), a well‐validated observational instrument administered at a few fixed timepoints [6]. Its validity is not in question. But validity at the moments it samples is a different property from completeness across the whole experience: the mYPAS captures discrete snapshots, and if distress is episodic—a spike at an unexpected handling event, a surge when a parent steps away, a reaction to an unfamiliar face—spikes that do not coincide with a scheduled assessment are invisible to standard care. The interventions exist; the timing information does not.

Continuous wearable physiology lets us examine both properties at once, and translating raw wearable data into a usable signal is itself instructive. We conducted a feasibility study of continuous wrist‐worn monitoring across the pediatric pre‐anesthetic period to test a twofold hypothesis: first, that the wearable heart‐rate signal would correlate with the mYPAS where it samples, establishing that the physiology carries genuine anxiety‐relevant information and that the instrument is doing its job; and second, that heart‐rate elevations would nonetheless occur largely outside those windows, establishing that a valid instrument can still be an incomplete one. We frame the work as a feasibility and signal‐characterization study, not a test of any intervention.

## 2 Materials and Methods

### 2.1 Design, Setting, and Oversight

Prospective single‐center feasibility study at a community‐based, rural‐serving pediatric surgical center. The study was approved by the UPMC Central Pennsylvania Region Institutional Review Board, which served as the IRB of record (protocol CPAR25‐001; initial approval 8 April 2025, approval current). Parental permission and child assent were obtained, with consent materials available in English and Spanish.

### 2.2 Participants

Children aged 4–17 scheduled for elective surgery requiring general anesthesia were eligible; emergency cases were excluded. The age range (4–17 years) was the prespecified protocol population representing the pediatric ambulatory surgical population; age 4 was not a lower limit of mYPAS validity. All participants underwent elective outpatient pediatric general‐surgical procedures, all were ASA physical status I, and none had prior surgical or anesthetic experience.

### 2.3 Device and Signals

Participants wore a wrist biosensor device (Empatica EmbracePlus) [7] from preoperative holding through induction. The data available for this analysis comprised a per‐minute series of heart rate (photoplethysmography‐derived, beats/min), EDA (μS), and skin temperature (°C). Two constraints bear on interpretation. First, although the device is capable of raw, higher‐rate signal acquisition, only per‐minute summaries were retained for this cohort; beat‐to‐beat inter‐beat intervals, raw photoplethysmography, accelerometry, and oxygen‐saturation data were not retained, so heart‐rate variability (HRV) [8] could not be computed or reconstructed from an averaged rate. Second, wrist EDA was flat or absent in 10 of 15 children. Heart rate was the analytic channel; channel‐level informativeness and detector‐referencing analyses from this cohort are reported in a companion methodological report [9]. The EmbracePlus/EpiMonitor system is FDA‐cleared for seizure detection using EDA and motion in individuals aged 6 and older; wrist photoplethysmographic pulse rate is a research signal that is not part of any cleared clinical output, and we report it as a research measurement rather than a validated clinical parameter.

### 2.4 Behavioral Assessment

mYPAS was administered at three timepoints whose context shapes interpretation: the first during consent (an active state rather than rest), the second at parental separation, and the third at induction. The end of each usable recording corresponded to induction. Midazolam premedication status and, where available, timing were recorded. In addition to the mYPAS, child self‐reported anxiety (State–Trait Anxiety Inventory for Children [STAIC], Form C‐1 state and Form C‐2 trait) and parental anxiety (State–Trait Anxiety Inventory short form, state and trait) were collected; child self‐report was analyzed only in children aged ≥ 9, the validated range for the instrument.

### 2.5 Concordance Analysis

We computed mean heart rate in the induction window and its Spearman correlation with induction anxiety (mYPAS3), tested the within‐patient change from earlier windows with the Wilcoxon signed‐rank test, and examined absolute heart‐rate concordance at the consent, settled‐holding, and induction windows. Because heart rate declines with age in children, age was examined as a potential confounder via a first‐order partial Spearman correlation of induction‐window heart rate with mYPAS3 adjusting for age, computed from the pairwise Spearman correlations of heart rate, mYPAS3, and age. Absolute heart rate was interpreted against age‐expected values (American Heart Association Pediatric Advanced Life Support reference ranges, 2015 edition: 80–120 bpm ages 3–5, 75–118 ages 6–11, 60–100 ages 12–17). Convergent validity was assessed as the Spearman correlation of induction‐window heart rate with child and parent self‐reported anxiety.

### 2.6 Heart‐Rate‐Elevation Detection and Coverage

Within each child's heart‐rate series we detected discrete heart‐rate elevations as local excursions (peak detection; minimum prominence 10 beats/min; minimum separation 3 min). For each event we determined whether it fell within ±5 min of any mYPAS timepoint (within‐window) or outside all of them (outside‐window), with sensitivity analyses at ≥ 8 and ≥ 12 beats/min (the latter with a ±3‐min window). Fourteen children with reliable timepoint timing were included, and the analysis was stratified by midazolam premedication.

### 2.7 Statistical Analysis

Analyses are exploratory; we report Spearman correlations (Fisher‐*z* confidence intervals), Wilcoxon signed‐rank and Mann–Whitney tests, and event proportions with child‐clustered bootstrap intervals, without correction for multiple comparisons. Heart rate was the sole analytic channel; analyses used Python (SciPy, NumPy, pandas, NeuroKit2).

### 2.8 Sample Size and Statistical Framing

This is a feasibility analysis of a fixed convenience sample, not a study sized to confirm a hypothesis, and we therefore do not report a power calculation. The cohort was determined by available enrollment, and post hoc power computed from observed effects would be circular and uninformative. Consistent with the purpose of a feasibility study, the analyses are exploratory and effect‐estimating rather than confirmatory: we report effect sizes with 95% confidence intervals so that their precision—which is limited at this sample size—is explicit, and we treat *p* values as descriptive. These estimates are intended to anchor the sample‐size calculation for a subsequent, adequately powered prospective study rather than to establish efficacy here. Correlation intervals use the Fisher z transformation; the outside‐window proportion is reported with a child‐clustered bootstrap interval (10 000 resamples) to account for multiple events per child.

## 3 Results

### 3.1 Cohort and Feasibility

Thirty‐two children consented to participate (Figure S1). Thirteen did not undergo monitoring: four subsequently required emergency surgery and nine declined participation on the day of surgery. Of the 19 children monitored, 4 did not yield an analyzable recording because the device was displaced in 2 and no usable signal was obtained in 2, leaving 15 children in the analytic cohort. These losses were logistical or technical rather than exclusions based on observed heart‐rate or mYPAS data; however, selection bias cannot be fully excluded. The 15 children with quality‐controlled, time‐aligned data (median age 10 years, range 4–16; nine male, six female; eight premedicated) form the analytic cohort. All underwent elective outpatient pediatric general‐surgical procedures, all were ASA physical status I, and none had prior surgical or anesthetic experience. Continuous wrist capture was feasible from holding through induction, with high heart‐rate coverage (median ~98% of minutes).

### 3.2 The Wearable Correlates With the Validated mYPAS

The wearable heart‐rate signal tracked behavioral anxiety at the moments the mYPAS samples, supporting that both modalities capture anxiety‐relevant autonomic activity. Heart rate rose from earlier windows into induction in 13 of 15 children (87%, 95% CI 60%–98%; median + 10 beats/min; Wilcoxon *p* = 0.022), the direction expected if induction is the period of acute autonomic activation. In the pre‐specified comparison, absolute induction‐window heart rate correlated with induction anxiety (mYPAS3) at Spearman *ρ* = 0.55 (95% CI 0.01–0.84; *p* = 0.034; *ρ* = 0.57 with the single out‐of‐range participant excluded)—a moderate concordance of the magnitude expected between two imperfect measures of the same underlying state, neither trivial nor implausibly strong.

Two features of this concordance shape how the signal should be used. First, the relationship is carried by the absolute heart‐rate level, not within‐patient change: absolute heart rate across the pre‐anesthetic windows tracked induction anxiety (consent‐window *ρ* ≈ 0.60; a settled mid‐holding window *ρ* ≈ 0.73; induction *ρ* = 0.55), whereas within‐patient change between windows correlated with anxiety at essentially zero (*ρ* ≈ 0). In this cohort, absolute heart‐rate level appeared more informative than within‐child change. Because heart rate falls with age in childhood, absolute level must be read against age, and we tested whether the concordance was merely an age effect: induction heart rate was strongly age‐dependent (*ρ* = −0.74) and anxiety more weakly so (*ρ* = −0.41, not significant), but the concordance persisted after adjustment for age (partial Spearman *ρ* = 0.46), although the estimate was imprecise in this small cohort. Second, the earlier‐window estimates are descriptive and were examined post hoc—the settled‐holding value in particular is fragile at this sample size and should be read as hypothesis‐generating, with the induction comparison as the anchor (Figure 1). The consistent direction across windows, together with the null for within‐patient change, is the methodologically important pattern, developed further in the companion methodological report [9]. This is the validation half of the picture: where the mYPAS looks, the physiology agrees with it.

**FIGURE 1:**
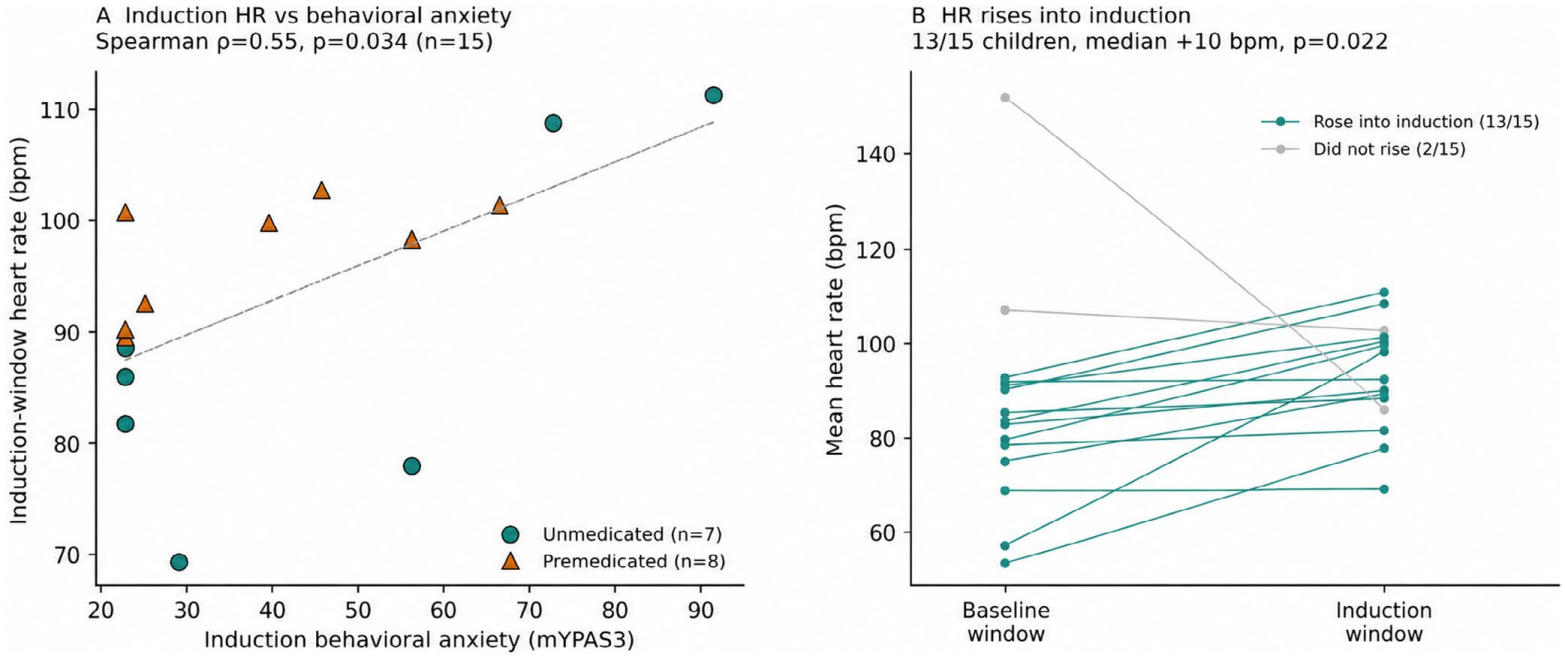
(A) Absolute induction‐window heart rate versus induction anxiety (mYPAS3); Spearman *ρ* = 0.55. (B) Within‐patient heart rate from baseline to induction, rising in 13 of 15 children (median +10 beats/min; *p* = 0.022); gray lines mark the two children whose heart rate did not rise. The wearable signal correlates with the validated instrument at the sampled moment.

### 3.3 Convergent Validity Across Informants

Because no single behavioral instrument is a complete ground truth, we asked whether the heart‐rate signal correlated with anxiety reported by the child and by the parent, not only with the observer‐rated mYPAS. Among children old enough for self‐report to be valid (ages ≥ 9, *n* = 9), self‐reported state anxiety (STAIC Form C‐1) correlated with induction‐window heart rate at Spearman *ρ* = 0.62 (95% CI −0.15 to 0.92; *p* = 0.075), whereas trait anxiety (Form C‐2) did not (*ρ* = 0.00). The physiology tracked how anxious a child reported feeling in the moment rather than their general disposition—the state‐specific pattern expected of a genuine situational (state) signal.

Parental anxiety showed no clear relationship with the child's physiology or behavior in this sample: parent state anxiety correlated weakly with induction heart rate (*ρ* = 0.30, *p* = 0.32) and negligibly with mYPAS3 (*ρ* = 0.14), with trait and enrollment measures similar. We report this as a null given the sample size, notwithstanding the parental‐anxiety transmission documented in larger cohorts [1]. The three informants agreed only modestly with one another (child self‐report vs. observer mYPAS *ρ* = 0.25; parent vs. child *ρ* = 0.23), consistent with the established view that observational, self‐report, and proxy measures capture overlapping but distinct facets of anxiety. That the continuous heart‐rate signal correlated with the two situational measures—observer mYPAS3 (*ρ* = 0.55) and child state self‐report (*ρ* = 0.62)—more closely than those measures agreed with each other supports its use as an objective adjunct that no single behavioral scale fully replaces. These self‐report analyses are exploratory, limited by sample size and by a compressed mYPAS3 range in the older subgroup.

### 3.4 Heart‐Rate Elevations Frequently Occur Between the Assessments

Viewing each child's entire recording reveals the limit of three snapshots. Heart‐rate elevations of undetermined cause appeared scattered throughout the pre‐anesthetic period, while the three mYPAS timepoints sampled only narrow slices of it (Figure 2). The assessment intervals varied: across the 14 children with reliable timing, the consent‐to‐separation interval (median 44.5 min; IQR 37–53.5; range 10–248) was longer than the separation‐to‐induction interval (median 6.5 min; IQR 5–8.75; range 4–14). Quantifying this across 14 children, 90 heart‐rate elevations were detected; only 26 (29%) fell within ±5 min of a mYPAS timepoint and 64 (71%; child‐clustered 95% CI 58%–80%) fell outside all assessment windows. Children had a median of 5 events each (range 1–22) while the three timepoints sample only three brief windows of the period (Figure 3); 12 of 14 children (86%; 95% CI 57%–98%) had ≥ 1 heart‐rate elevation outside all assessment windows. At the patient level, children had a median of 4 outside‐window elevations each (IQR 2–6; 3.2 per hour), and excluding the single child with 22 elevations, the pooled outside‐window proportion was essentially unchanged (65% across the remaining 13 children). The result was robust to detection settings: 73% of elevations fell outside all windows at a lower threshold (≥ 8 beats/min) and 85% under a stricter definition (≥ 12 beats/min, ±3‐min window). Because the ±5‐min window is generous and the recording spans only holding through induction, 71% is a conservative estimate of the outside‐window proportion across the full perioperative trajectory.

**FIGURE 2:**
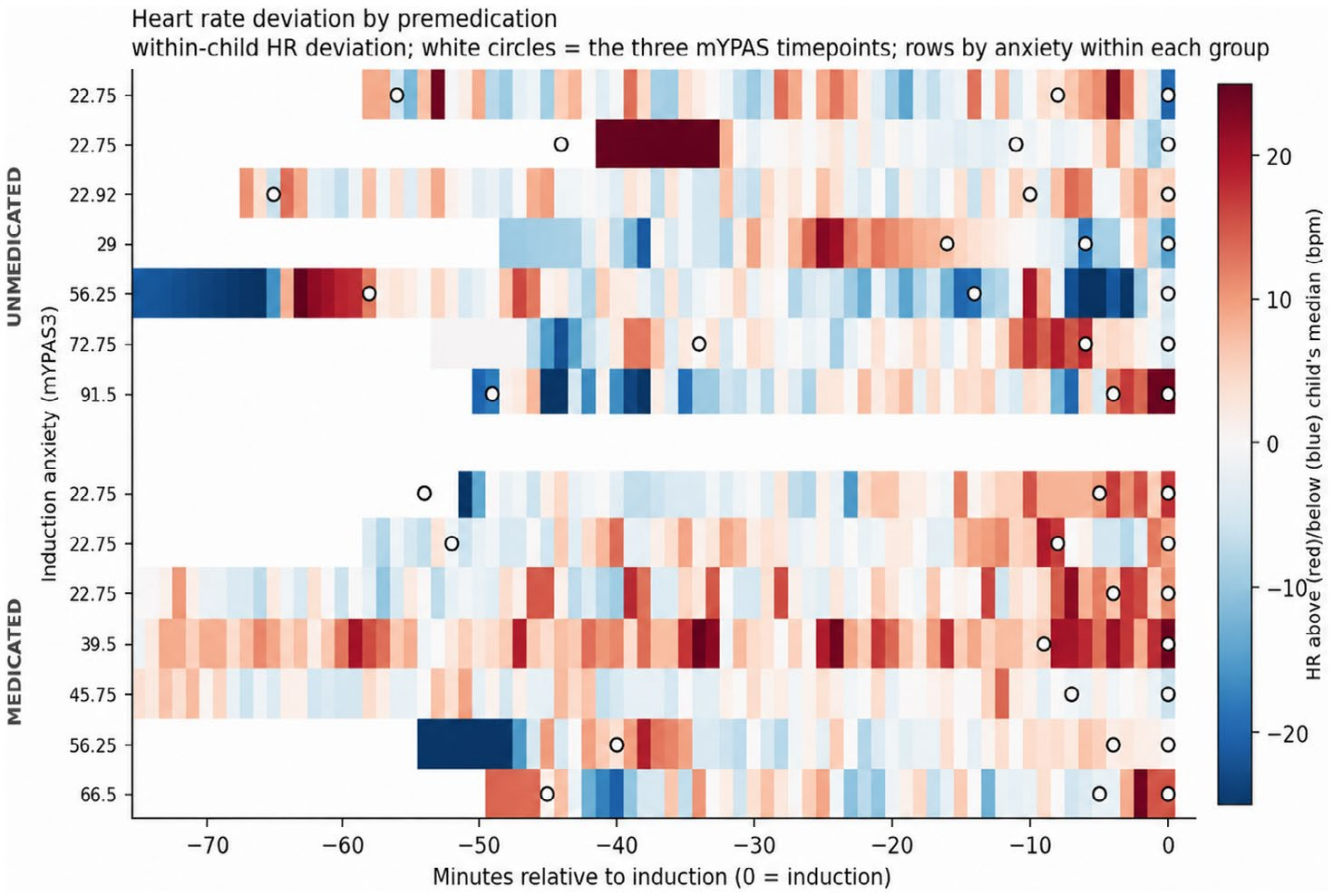
The whole cohort, divided by premedication. Each row is one child's heart rate over the period before induction, colored by deviation above (red) or below (blue) that child's own median (within‐child scaling for cross‐age comparability; a display choice distinct from the absolute‐level analysis). White circles mark the three mYPAS timepoints. Rows are ordered by induction anxiety within each group. Red elevations appear throughout each recording while the three sampled moments capture little of it, in medicated and unmedicated children alike. The saturated early block in one unmedicated child is a movement/placement artifact (see Section 4.2). Two recordings extend well beyond the window shown owing to surgical delay.

**FIGURE 3:**
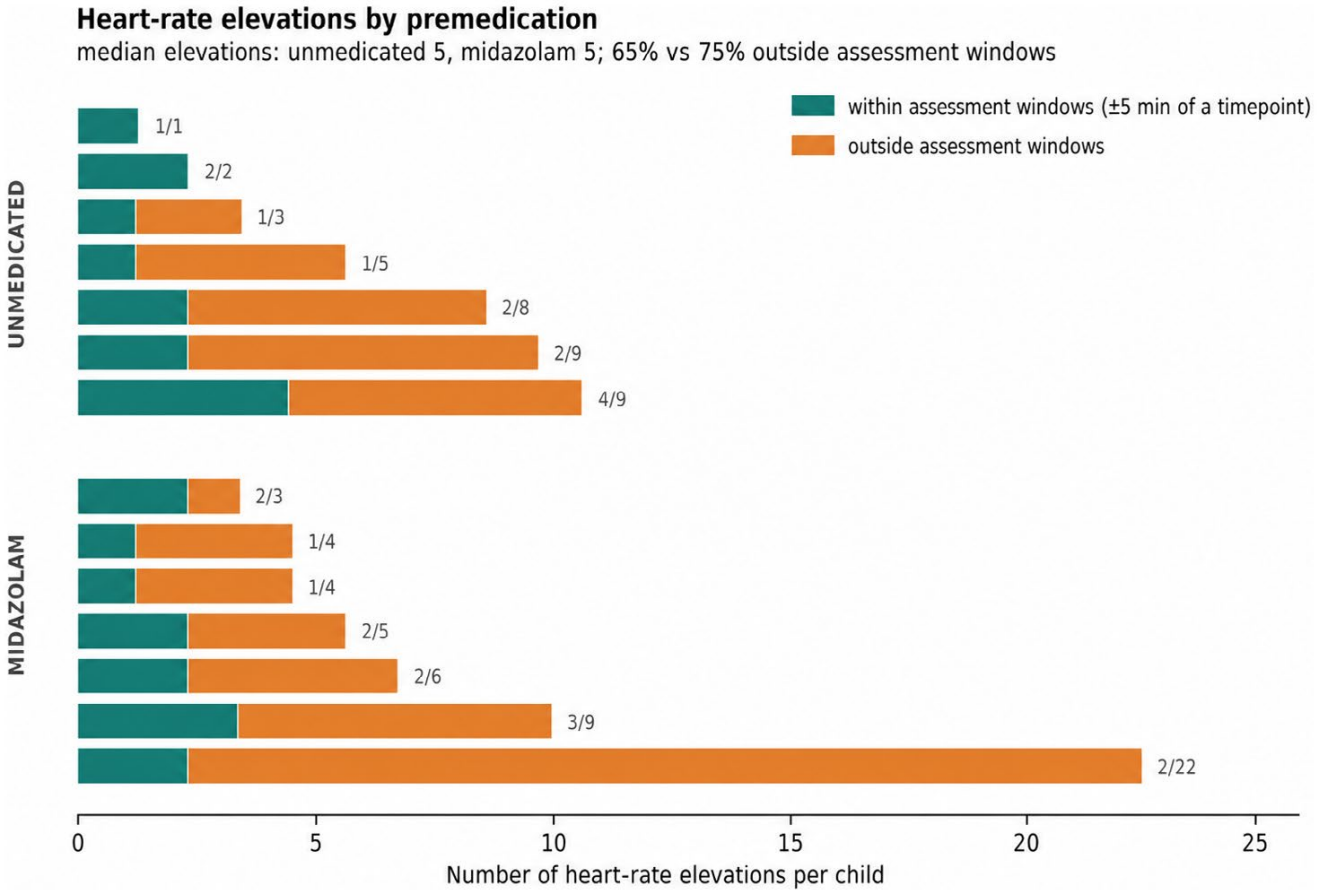
Heart‐rate elevations per child, divided by premedication. Bars show the number of elevations falling within (teal) and outside (orange) the three assessment windows; most elevations fell outside all windows in both groups.

### 3.5 Premedication Did Not Eliminate the Outside‐Window Pattern

Midazolam did not reduce the number of heart‐rate elevations (median 5 per child in both groups; events/h 3.7 unmedicated vs. 4.1 medicated; *p* = 0.71), and the outside‐window pattern was present in both (65% of elevations outside windows unmedicated, 75% medicated; Figures 2 and 3). Premedicated children had higher median induction heart rate and mYPAS3 scores (100 vs. 86 beats/min; mYPAS3 39.5 vs. 29), but this is best read as confounding by indication and age—clinicians premedicate the children expected to be anxious, and younger children are both more often premedicated and higher‐heart‐rate—rather than a drug effect; at *n* = 7 per group no between‐group comparison was significant, and midazolam timing was heterogeneous.

### 3.6 Negative and Null Findings

Heart rate at the separation timepoint did not correlate with separation anxiety (*ρ* = 0.25), and separation was not the behavioral peak in this cohort (mYPAS2 was the highest of the three scores in only 7 of 14 children)—compatible with midazolam attenuating separation distress, though the cohort was too small to confirm.

## 4 Discussion

Two findings sit side by side. First, the wearable heart‐rate signal correlated with the mYPAS where the mYPAS samples—heart rate rose into induction in nearly every child and absolute induction heart rate tracked induction anxiety—supporting that the instrument captures real autonomic activity and that our signal captures the same thing. The mYPAS is doing its job. Second, that same continuous signal shows the instrument cannot, by construction, capture most of what happens: roughly 71% of heart‐rate elevations fell outside its three assessment windows, and 12 of 14 children had heart‐rate elevations that no scheduled assessment observed. The conclusion is not that the mYPAS is inaccurate, but that it is incomplete—accurate where it samples, and uninformative about the intervals between samples.

The clinical consequence is about timing. Effective tools for pediatric distress already exist, but they are deployed on a fixed schedule and clinician judgment rather than in response to continuously measured physiologic variation, because that variation is largely unscheduled and not concurrently observed. Variation occurring outside the sampled windows is not captured by fixed‐timepoint assessment. Continuous physiologic monitoring adds information about heart‐rate variation in the intervals between assessments; whether that variation reflects anxiety, and whether it could inform the timing of intervention, is untested here and would require concurrent behavioral measurement. The same pattern was present in premedicated children.

### 4.1 Relation to Prior Work

Wearable stress monitoring has been studied extensively in adults, but a recent scoping review found only a small minority of machine‐learning studies enrolled children, and none addressed a perioperative setting [10]. The mYPAS itself has been validated chiefly against other behavioral instruments rather than against physiology [11]. Where physiologic measures and the mYPAS have been recorded together—for example heart rate during pediatric MRI preparation [12], or vital signs in distraction and premedication trials—the two have generally been reported as separate or parallel outcomes rather than correlated within children against the mYPAS. Prior pediatric perioperative autonomic work, in turn, has relied on electrocardiographic measures in narrow surgical contexts without concurrent behavioral assessment [13]. To our knowledge, this is among the first studies to correlate continuous wrist‐worn physiology with the mYPAS at the level of individual assessments and to quantify the instrument's temporal incompleteness in children.

### 4.2 Limitations

A heart‐rate elevation was defined operationally from the recorded signal, but its physiologic and behavioral cause cannot be determined from this study; an excursion may reflect movement, an intravenous catheter, mask application, crying, environmental stimuli, or artifact, and elevations outside assessment windows lack concurrent behavioral reference. The sample is small and single‐center; the device exported only minute‐aggregated data, precluding HRV, and wrist EDA was largely uninformative. Wrist photoplethysmographic pulse rate is not an FDA‐cleared clinical output and is reported here as a research measurement; we did not identify device‐specific validation of wrist‐PPG pulse‐rate accuracy in children, and a motion‐resistant pulse‐oximetry system may be more practical when the intended output is pulse rate alone. The present analysis relied on minute‐averaged pulse rate and did not demonstrate an advantage over simpler continuous pulse‐rate monitoring; comparative signal‐quality testing was not performed. Age‐dependent normal heart rate, developmental differences in mYPAS behaviors, and poorer wrist fit or greater motion artifact in smaller children are additional limitations. The heart‐rate‐elevation threshold is an analytic choice, mitigated but not eliminated by the robustness checks. The recording spanned only holding through induction, making the coverage estimate a lower bound. The consent‐coincident baseline and prevalent midazolam use are confounders a purpose‐built study should annotate and model. Procedure‐specific diagnoses and operations are not reported because of the potential for re‐identification in this small single‐center cohort; consequently, the possible influence of procedure type on preoperative anxiety could not be evaluated.

### 4.3 Future Directions

Establishing whether these heart‐rate elevations represent distress requires continuous or event‐locked behavioral ground truth—time‐synchronized video‐coded distress or trained‐observer annotations—with an accelerometer channel for motion and a device that streams raw signal to recover HRV and usable EDA. Capture should extend across the full perioperative trajectory; separation and other events should be precisely timestamped; premedication timing should be modeled as a time‐varying covariate; and analyses, including the exploratory observation that settled preoperative tone may predict induction anxiety, should be pre‐registered.

## 5 Conclusion

Continuous wrist‐worn heart‐rate monitoring correlates with the validated mYPAS yet shows that heart‐rate elevations of undetermined cause occur between its assessments. The mYPAS is accurate but not complete; continuous monitoring adds information about heart‐rate variation between assessments; whether this can improve the timing of interventions requires prospective testing with concurrent behavioral measurement. These findings suggest that behavioral observation and continuous physiology answer different questions rather than competing ones: the mYPAS accurately measures anxiety when it is applied, whereas continuous monitoring characterizes the intervals between observations.

## Author Contributions

**Shawn D. Safford:** conceptualization, methodology, investigation, resources, data curation, formal analysis, funding acquisition, project administration, supervision, writing – original draft, writing – review and editing. **Sikiru Adewale:** formal analysis, software, data curation, validation, visualization, writing – original draft, writing – review and editing. **Denis Gracanin:** conceptualization, methodology, formal analysis, supervision, funding acquisition, writing – original draft, writing – review and editing.

## Funding

This study was supported by a Pennsylvania Medical Society Innovation Grant, which had no role in study design, data collection and analysis, interpretation of the data, or the decision to submit for publication.

## Ethics Statement

Approved by the UPMC Central Pennsylvania Region Institutional Review Board (protocol CPAR25‐001); parental permission and child assent obtained.

## Conflicts of Interest

The authors declare no conflicts of interest.

## Supporting information


**Figure S1:** Participant flow. Of 32 children who consented, 13 did not undergo monitoring (emergency surgery, *n* = 4; declined participation on the day of surgery, *n* = 9) and, of the 19 monitored, 4 did not yield an analyzable recording (device displaced, *n* = 2; no usable signal, *n* = 2), yielding 15 children with analyzable, time‐aligned data. Attrition reflected logistical or technical factors rather than selection on observed data; selection bias cannot be fully excluded.

**Data S1:** STROBE checklist.

## Data Availability

De‐identified per‐minute data and analysis code are available on reasonable request.
