# Supplementary material for: Continuous Wrist‐Derived Heart Rate Correlates With mYPAS Scores and Detects Between‐Assessment Heart‐Rate Elevations in Children: A Feasibility Study
Source: Paediatr Anaesth. 2026 Sep 10;36(12):1571–7. doi: 10.1002/pan.70309 (PMC13748701; doi:10.1002/pan.70309)
Supplement: Supplementary file 1 [file PAN-36-1571-s001.docx]

**STROBE Checklist — Supporting Information**

*Continuous Wrist Heart Rate Correlates With mYPAS Scores Yet Reveals Arousal Between Assessments in Children: A Feasibility Study*

| **Item** | **STROBE recommendation** | **Location in manuscript** |
| --- | --- | --- |
| ***Title and abstract*** | | |
| 1 (a) | Indicate the study’s design with a commonly used term in the title or abstract | Title; Abstract |
| 1 (b) | Provide an informative and balanced summary in the abstract | Abstract (structured: Background, Aims, Methods, Results, Conclusions) |
| ***Introduction*** | | |
| 2 | Background and rationale for the investigation | Introduction (§1) |
| 3 | State specific objectives, including any prespecified hypotheses | Introduction (§1); Abstract (Aims) |
| ***Methods*** | | |
| 4 | Present key elements of study design early in the paper | Methods §2.1 (single-center prospective observational feasibility study) |
| 5 | Setting, locations, and relevant dates | Methods §2.1; Declarations (IRB approval/dates) |
| 6 | Eligibility criteria; sources and methods of selection of participants | Methods §2.2; Results §3.1; Figure S1 (flow) |
| 7 | Clearly define outcomes, exposures, predictors, and potential confounders | Methods §2.4 (mYPAS, child/parent self-report), §2.5 (heart-rate concordance; age as confounder), §2.6 (arousal events) |
| 8 | For each variable, give sources of data and measurement methods | Methods §2.3 (wrist biosensor), §2.4 (behavioral instruments) |
| 9 | Describe any efforts to address potential sources of bias | Methods §2.5 (age-adjusted partial correlation); Results §3.1 (attrition logistical/technical, not selective); Discussion §4.2 |
| 10 | Explain how the study size was arrived at | Methods §2.8 (feasibility convenience sample; no power calculation; effect-estimating) |
| 11 | Explain how quantitative variables were handled in the analyses | Methods §2.5–§2.6 (absolute heart rate; arousal-event detection parameters) |
| 12 | Statistical methods, including those for confounding, subgroups, missing data, and sensitivity analyses | Methods §2.5, §2.7 (Spearman, Wilcoxon, Mann-Whitney; child-clustered bootstrap; exploratory, uncorrected) |
| ***Results*** | | |
| 13 | Numbers of individuals at each stage; reasons for non-participation; flow diagram | Results §3.1; Figure S1 (participant flow) |
| 14 | Characteristics of study participants and information on missing data | Results §3.1 (age, sex, premedication; heart-rate coverage) |
| 15 | Report outcome events or summary measures | Results §3.2–§3.6 |
| 16 | Main results with estimates and their precision (e.g., 95% CI) | Results §3.2 (concordance), §3.3 (convergent validity), §3.4 (coverage gap) — all with 95% CIs |
| 17 | Other analyses — subgroups, interactions, sensitivity analyses | Results §3.5 (premedication strata), §3.2 (age adjustment), §3.4 (outlier sensitivity) |
| ***Discussion*** | | |
| 18 | Summarise key results with reference to study objectives | Discussion §4 (opening) |
| 19 | Limitations, accounting for sources of bias or imprecision | Discussion §4.2 |
| 20 | Cautious overall interpretation considering objectives, limitations, and other evidence | Discussion §4, §4.1 |
| 21 | Generalisability (external validity) of the results | Methods §2.1 (community-based, rural-serving center); Discussion §4.1 |
| ***Other information*** | | |
| 22 | Source of funding and the role of funders | Declarations (Funding) |

**Note.** Adapted from the STROBE Statement checklist for observational (cross-sectional) studies. Section numbers refer to the main manuscript; page and line numbers will be added against the typeset proof at submission.
