# Supplementary material for: Continuous Wrist‐Derived Heart Rate Correlates With mYPAS Scores and Detects Between‐Assessment Heart‐Rate Elevations in Children: A Feasibility Study
Source: Paediatr Anaesth. 2026 Sep 10;36(12):1571–7. doi: 10.1002/pan.70309 (PMC13748701; doi:10.1002/pan.70309)
Supplement: Supplementary file 2 [file PAN-36-1571-s002.docx]

**Supporting Information**

The following are available online as Supporting Information for this article.

**Figure S1 — Participant flow. Of 32 children who consented, 13 did not undergo monitoring (emergency surgery, n = 4; declined participation on the day of surgery, n = 9) and, of the 19 monitored, 4 did not yield an analyzable recording (device displaced, n = 2; no usable signal, n = 2), yielding 15 children with analyzable, time-aligned data. Attrition reflected logistical or technical factors rather than selection on observed data; selection bias cannot be fully excluded.**
